# Fingolimod synergizes with anti-PD-1 radioimmunotherapy in an experimental multiple sclerosis model through enhanced lymph node retention and CD8^+^ T cell depletion

**DOI:** 10.3389/fmed.2025.1593933

**Published:** 2025-07-03

**Authors:** Connor Frank, Kevin J. H. Allen, Wojciech Dawicki, Michael C. Levin, Ekaterina Dadachova

**Affiliations:** ^1^College of Pharmacy and Nutrition, University of Saskatchewan, Saskatoon, SK, Canada; ^2^Department of Biochemistry, Microbiology and Immunology, College of Medicine, University of Saskatchewan, Saskatoon, SK, Canada; ^3^Office of the Saskatchewan Multiple Sclerosis Clinical Research Chair, University of Saskatchewan, Saskatoon, SK, Canada; ^4^Cameco MS Neuroscience Research Centre, College of Medicine, University of Saskatchewan, Saskatoon, SK, Canada; ^5^Neurology Division, College of Medicine, University of Saskatchewan, Saskatoon, SK, Canada; ^6^Department of Anatomy, Physiology and Pharmacology, College of Medicine, University of Saskatchewan, Saskatoon, SK, Canada

**Keywords:** nuclear medicine, radioimmunotherapy, multiple sclerosis, autoimmunity, theranostic, SPECT/CT

## Abstract

**Introduction:**

Multiple sclerosis (MS) is a chronic neurodegenerative autoimmune disease caused by inappropriate activation of the immune system that targets central nervous system antigens, such as myelin. Some MS therapies control the disease through the depletion of CD20+ B cells and the sequestration of T lymphocytes in lymph nodes. A targeted approach aimed at depleting only activated T lymphocytes, which play a key role in disease progression, would be optimal. Programmed cell death protein 1 (PD-1) is an inhibitory receptor expressed on exhausted and recently activated T cells. We have previously reported success with [^177^Lu]Lu-anti-PD-1 (^177^Lu-αPD-1) radioimmunotherapy (RIT) as a standalone treatment in experimental autoimmune encephalomyelitis (EAE), which is the most common preclinical model of MS. In this study, we present the synergistic effect of fingolimod (FTY720/Gilenya™) in combination with [^177^Lu]Lu-αPD-1 in EAE.

**Methods:**

[^177^Lu]Lu-αPD-1 and [^111^In]In-αPD-1 were prepared by conjugating a commercial anti-murine PD-1 antibody with a DOTA bifunctional chelating agent. Therapy using [^177^Lu]Lu-αPD-1 ± FTY720 was initiated at the EAE symptom onset in MOG 35–55 immunized C57Bl/6 mice. ImmunoSPECT/CT imaging utilizing [^111^In]In-αPD-1 was performed with or without FTY720 administration at the symptom onset in EAE mice. Flow cytometry was performed on spinal cord mononuclear cells isolated from EAE mice 7 days post treatment with RIT ± FTY720.

**Results:**

[^177^Lu]Lu-αPD-1 in combination with FTY720 administration significantly reduced long-term paralysis in EAE mice. ImmunoSPECT/CT imaging revealed enhanced lymph node retention of [^111^In]In-αPD-1 antibody in conjugation with FTY720, corresponding to the presence of activated lymphocytes in lymph nodes. Flow cytometry performed on isolated spinal cord mononuclear cells demonstrated a significant reduction in CD8^+^ T cell counts in the spinal cords of animals treated with [^177^Lu]Lu-αPD-1 and [^177^Lu]Lu-αPD-1 + FTY720. Immunofluorescent microscopy of thoracic spinal cord sections from treated animals demonstrated reduced demyelination and moderate infiltration of CD3^+^ lymphocytes in the [^177^Lu]Lu-αPD-1 + FTY720 group at study termination.

**Conclusion:**

[^177^Lu]Lu-αPD-1-targeted radioimmunotherapy was synergistically enhanced utilizing the approved MS therapeutic FTY720 through increased lymph node retention and irradiation of activated T lymphocytes. This study paves the way for the application of PD-1+ T cell-targeted radioimmunotherapy as a potential theranostic agent for MS.

## Introduction

Multiple sclerosis (MS) is a chronic inflammatory neurodegenerative disease affecting the central nervous system (CNS). Inflammation in the CNS is mediated by dysfunctional T and B cells, which are likely activated against antigens that cross-react with self-antigens found in the CNS, such as myelin oligodendrocyte protein (MOG) or proteolipid protein (PLP). This has been observed in murine models and human MS patients (1–3). MS patients experience neurological relapses that are associated with the acute onset of inflammation, followed by a period of remission described as “relapsing–remitting MS (RRMS),” with this cycle continuing throughout long periods of their lifetime (4). Over time, CNS symptoms can worsen without relapses, leading to the loss of the relapsing–remitting phenotype and the development of progressive disease. RRMS is considered to be dominated by T cell-mediated responses, while progressive forms of MS are mediated by the development of meningeal and CNS-adjacent B-cell clusters and “smoldering” lesions that remain active for years (5). These lesions are characterized by the formation of B-cell leptomeningeal clusters located adjacent to the white matter of the CNS. These B-cell leptomeningeal clusters are associated with axon loss and demyelination in addition to the secretion of anti-self-antigen antibodies within the CNS of MS patients (6, 7).

The initial waves of inflammation associated with the development of MS are coordinated in part by CD4^+^ T helper (Th) cells. Two subtypes of CD4^+^ T cells that are crucial for the pathogenesis of MS include interleukin-17 (IL-17)-producing Th17 and interferon-y (IFN-y)-producing Th1 cells (1). These CD4^+^ subsets have been identified as major components of MS CNS lesions and are required for the development of MS-like disease in murine models (8). A crucial step in activating CD4^+^ T cells is the interaction between the T cell receptor (TCR) and human leukocyte antigen (HLA) in humans, or between TCR and major histocompatibility complex (MHC) in mice. MHC molecules carry antigen fragments that are presented on professional antigen-presenting cells (APCs) to the TCR on CD4^+^ T cells (9). Upon TCR stimulation, T cells upregulate various co-stimulatory and co-inhibitory molecules to maintain homeostatic balance and regulate overactivation. A well-established co-inhibitory molecule is the programmed cell death protein 1 (PD-1), which is a negative regulator of T cell activation (10). Upon TCR-mediated stimulation, T cells rapidly upregulate PD-1 to the cell surface. In normal circumstances, PD-1 interacts with its cognate ligands, PD-L1 (B7-H1) or PD-L2 (B7-H2), on APCs. PD-1 is not expressed on naïve, non-TCR stimulated CD4^+^ and CD8^+^ T cells in the periphery (11). In autoimmune diseases, PD-1^+^ cells have been identified in the CNS tissues of EAE-induced mice, which is an animal model of MS (12). Other studies have demonstrated that CNS-infiltrating CD4^+^ T cells express PD-1 during the onset of acute EAE (13).

Fingolimod (FTY720/Gilenya™) is a sphingosine-1-phosphate receptor-1 (S1PR1) antagonist. FTY720 inhibits S1P1R-mediated egress of T cells from the lymph node to peripheral tissue (14). The interaction between APC and T cells occurs in the lymph node, and after antigen-mediated TCR stimulation, T cells migrate to the periphery to execute their functions. Inhibiting the T cell movement from the lymph node reduces CNS tissue damage and T cell-mediated inflammation in MS. FTY720 has demonstrated remarkable clinical success in controlling relapses in RRMS through its inhibition of T cell responses (15).

Radioimmunotherapy (RIT) is a targeted radiation approach utilizing an immune vector, such as a monoclonal antibody, to deliver cytocidal radiation to targeted cells (16). In contrast to traditional radiation therapy, RIT utilizes the specific targeting of the biological vector to precisely deliver potent radiation to the desired target. Minimizing off-target irradiation and total body absorbed dose reduces damage to adjacent cells and healthy tissue. Utilizing various medical radioisotopes in RIT allows the modulation of delivered dose and, importantly, dose rate. Recent clinical trials utilizing the beta particle (*β*^−^) emitting Lutetium-177 (^177^Lu), with a half-life of 6.7 days, led to the approval of two new radiopharmaceuticals [Pluvicto (17), Lutathera (18)]. The short length (~670 μm) emission of beta particles from ^177^Lu induces cell death through the ionization of DNA, cell membrane lipid components, and intracellular generation of reactive oxygen species (19, 20). An advantage of ^177^Lu is its imageable gamma ray emissions at 208 and 113 keV, which allows treatment via beta irradiation and monitoring via gamma ray detection in nuclear imaging. This approach allows dual treatment and diagnosis/imaging, which is referred to as theranostics.

We previously demonstrated that a ^177^Lu-labelled monoclonal antibody targeting the PD-1 receptor showed positive disease suppression in EAE (21). We also observed retention of the targeted RIT agent in the lymph nodes of EAE during immunoPET/CT imaging. In this study, we sought to combine the lymph node retention properties of FTY720 with radioablation of activated lymphocytes using a radiolabeled anti-PD-1 monoclonal antibody. We hypothesized that FTY720 would enhance the ability of [^177^Lu]Lu-αPD-1 to target and deplete autoreactive T cells. Reduction in activated T lymphocytes would alleviate symptoms and delay disease progression in an animal model of MS. We also sought to further understand the mechanism of action of this novel combination through analyzing isolated mononuclear cells and conducting immunohistochemistry of spinal cords of animals treated with RIT alone or in combination with FTY720.

## Materials and methods

### Antibody conjugation and radiolabeling

Rat IgG2a anti-mouse PD-1 monoclonal antibody (BioXCell, Clone: RMP1-14) was conjugated to the bifunctional chelator p-SCN-Bn-DOTA (Macrocyclics, Cat#B-205) using previously described procedures (21).

Non-carrier added Lutetium-177 chloride was provided by McMaster University (Hamilton, On, Canada). Indium-111 chloride (^111^In) was obtained from BWX Technologies (Lynchburg, Virginia, USA). Briefly, ^177^Lu or ^111^In was reacted with a conjugated antibody at a 1:10 μg/uCi ratio (1:0.37 μg/Mbq) in chelexed 0.15 M ammonium acetate solution. The reaction mixture was heated to 37°C for 1 h with shaking. It was then stopped by adding excess 5 mM EDTA solution. The reaction mixture was analyzed by pipetting a 1 μL of the sample from the bottom of an instant thin layer chromatography (iTLC) strip, using 0.15 M ammonium acetate as a mobile phase. After allowing the mobile phase to reach the top of the strip, the iTLC strips were read for 60 s on a BioScan AR-2000 imaging scanner (Eckert & Ziegler, Berlin, Germany). The iTLC strip was cut in half, with the mAb-radio complex retained at Rf = 0 and the free radioisotope at Rf = 3. Each half of the iTLC was then removed and analyzed separately on a Perkin Elmer Wizard 2 Gamma Counter (Perkin Elmer, Shelton, Connecticut, USA). Radiolabeling yield was calculated using the following equation:

$$\text{Radiochemical Yield}\;(\%)=\frac{\mathrm{CPM}\;\mathrm{Rf}=0}{\left(\mathrm{CPM}\;\mathrm{Rf}=0)+(\mathrm{CPM}\;\mathrm{Rf}=3\right)}*100.$$

Radioimmunoconjugates were confirmed to have a yield of >95% and were prepared in 0.9% sterile phosphate-buffered saline (PBS) for injection. RadioHPLC was performed using an Agilent AdvanceBio SEC 200A column (1.9 μm, 4.6 × 300 mm) on an Agilent 1260 Infinity II HPLC (Agilent Technologies, Santa Clara, California, USA). The radioimmunoconjugates were run in a 150 mM phosphate buffer (pH 6.9) at a flow rate of 0.35 mL/min for 20 min.

### EAE mouse model

Animal experiments were conducted in accordance with the guidelines set forth by the Canadian Council for Animal Care and were approved by the Ethics Review Board of the University of Saskatchewan Animal (Animal Use Protocol: 20200008). Experimental autoimmune encephalomyelitis (EAE) was induced following the methods previously described in the literature (21, 22). Nine-week old female C57Bl/6 mice (Charles River Laboratories, Strain #027) were injected subcutaneously with 100 µL in the upper and lower back of myelin oligodendrocyte glycoprotein residues 35-55 (MOG^35-55^) emulsified in complete Freund’s adjuvant (Hooke laboratories, Cat# EK-2110). The mice were then intraperitoneally injected twice with 110 ng of pertussis toxin in sterile PBS immediately following immunization and 24 h post-immunization. Symptoms of EAE were assessed on a standardized scale ranging from 0 to 5 as follows: (1) limp tail, (2) single leg hind paralysis or abnormal gait, (3) total hind paralysis or frontal limb paralysis, (4) total hind paralysis and frontal limp paralysis, and (5) moribund or dead animal. Animals euthanized due to severe paralysis corresponding to a score of 4 for >2 days were assigned a score of 5 for the remaining duration of the study. Animals that did not develop EAE, characterized by a maximal score ≤0.5 and the development of ulcerated lesions, were excluded from the study.

### Study design

**Figure 1:**
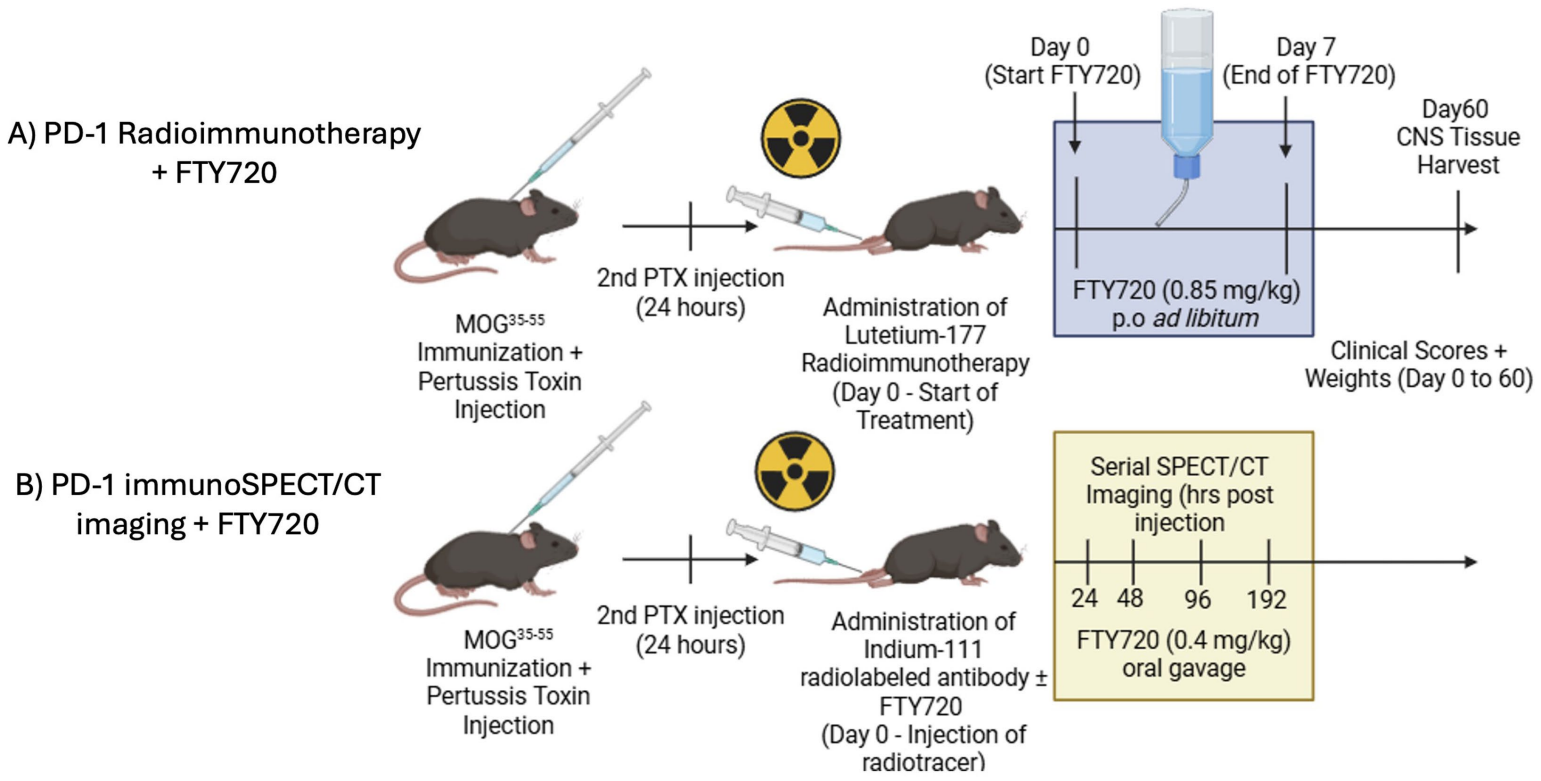
**(A)** Study design for combination therapy of anti-αPD-1 RIT + FTY720. **(B)** Study design for serial immunoSPECT/CT imaging of FTY720 administration.

We sought to establish a therapeutic design that would demonstrate synergy between T cell-targeted RIT utilizing FTY720, without depending on its immunomodulatory effects. Our study involved a short-term treatment of FTY720 in combination with ^177^Lu-αPD-1 (Figure 1A). We decided to initiate FTY720 treatment in drinking water rather than through daily oral gavage to minimize stress, which is a known confounding factor in EAE therapy, as increased levels of animal stress can suppress disease activity. To establish targeting and visualize T cell activity, we performed a similar ImmunoSPECT imaging study that involved daily administration of FTY720 via oral gavage to ensure equal dosing across animals for comparative purposes (Figure 1B).

### Combination of RIT and FTY720 treatment in EAE mice

For treatment studies, animals were randomized at the symptom onset (score = 0.5–1, flaccid or tip of tail weakness) to receive one of the following treatments: (1) saline vehicle, (2) [^177^Lu]Lu-αPD-1 (i.v., 2.22MBq), (3) [^177^Lu]Lu-αPD-1 (i.v., 2.22 MBq) + FTY720 (p.o., 0.8 mg/kg), or (4) FTY720 (p.o., 0.8 mg/kg) alone. Fingolimod hydrochloride (FTY720) was obtained from MedChemExpress (Cat# HY-12005) and prepared as a sterile solution in deionized water (ddH_2_0). FTY720 was then administered through drinking water (4 μg/mL). Animals were monitored daily and scored by treatment-blinded personnel. Non-radiolabeled anti-PD-1 control demonstrated no significant effect on the control of EAE, as described previously (21).

### ImmunoSPECT and image analyses

Animals were randomized at EAE symptom onset to receive one of the following treatments: (1) [^111^In]In-αPD-1 (i.v, 7.4 Mbq), (2) [^111^In]In-αPD-1 (i.v, 7.4 Mbq) + FTY720 (p.o, 0.4 mg/kg), (3) [^111^In]In-Control mAb (i.v, 7.4 Mbq), or (4) [^111^In]In-Control mAb (i.v, 7.4 Mbq) + FTY720 (p.o, 0.4 mg/kg). FTY720 was administered via oral gavage daily at a dose of 0.4 mg/kg in distilled water. Before imaging, animals were initially anesthetized with 5% isoflurane (1 L/min O_2_), received eye ointment, and a subcutaneous injection of 100 μL of 0.9% saline. Animals were then imaged at 24, 48, 96, and 192 h post radiotracer injection on a heated multi-rodent bed on a MiLABS Vector 4 SPECT/CT imager using a high-energy ultra-high resolution rat mouse (HE-UHR-RM) collimator (MiLabs, Utrecht, the Netherlands). Anesthesia was maintained with 2.5% isofluorane (1 L/min O_2_) for a 4-min CT scan (55 kV, 0.37 mA) and a 20-min SPECT acquisition collection on ^111^In energy emissions (171 and 245.4 keV). Images were then reconstructed using Milabs Reconstruction 8.00 RC6 software, using a pixel-based algorithm with 16 subsets, 10 iterations, and a voxel size of 0.12 mm^2^.

Region of interests (ROI) were drawn from the cervical draining lymph nodes, upper and lower back emulsion lesions, heart, liver, femur, intestines, kidneys, and brain using reconstructed CT and SPECT data on 3D Slicer v5.6.2. RTSS DICOM files were exported and analyzed in pMOD software (version 3.910, pMOD Technologies) to obtain total radioactivity or SUV/bw in the ROI (kBq).

### Flow cytometry

Spinal cord homogenates were processed for flow cytometry as previously described (21). Briefly, mononuclear cell isolates from the spinal cord tissue were obtained via 22% Percoll gradient centrifugation. Isolated cells were washed 3x with sterile PBS and stained with 1:1000 PBS diluted LIVE/DEAD™ Fixable Near IR 780 (Invitrogen, Cat# L34993). The samples were washed three times with PBS + 2% FBS + 0.02% sodium azide (FACS Buffer) and Fc blocked using a 1:100 dilution of anti-CD16/CD32 Fc block (Invitrogen, Clone: 93, Cat# 14-0161-82). Then, the samples were washed three times with FACS buffer and then stained with 1:100 diluted primary antibodies: anti-mouse CD45-PE (eBioscience, Clone: 30-F11), anti-mouse PD-1-Brilliant Violet 605 (BioLegend, Clone: 29F.1.A12), anti-mouse CD11b-eFluor 450 (eBioscience, Clone: M1/70), anti-mouse CD4-FITC (eBioscience, Clone: RM4-5), and anti-mouse CD8-PerCP/Cyanine5.5 (BioLegend, Clone: 53-6.7) for 1 h at 4°C. Finally, the samples were washed, and data were collected using a Beckman Coulter CytoFLEX, with analysis performed on FlowJo Software (ver.10.800).

T-distributed stochastic neighbor embedding (t-SNE) analysis was performed using the t-SNE FlowJo plugin. Gated, cleaned samples (Live, single cells) were analyzed through compensated fluorescent channels (CD45, CD11b, CD4, CD8, and PD-1) with opt-SNE learning configurations. Data were processed using 1,000 iterations, perplexity 30, learning rate 2,100, KNN algorithm exact, and Barnes–Hut gradient algorithm. Data plots are presented with color-coded gated populations (myeloid, microglia, lymphocyte, CD4 and CD8 T cells).

### Immunohistochemistry

Animals were deeply anesthetized using isoflurane and transcardially perfused with 30 mL of saline and 40 mL of fresh 4% paraformaldehyde. Tissues were then harvested, fixed in 4% paraformaldehyde overnight, and placed in a 15% sucrose solution until they sank. They were then moved to a 30% sucrose solution until the tissue sank. These tissues were then embedded in the OCT medium, snap-frozen using liquid N_2_-cooled isopentane for 15 s, and stored at −80°C until cryosectioning. The OCT-embedded tissues were cryosectioned on a Leica CM1850 cryostat at a thickness of 10 μm with a temperature of −20°C. The tissue sections were thenplaced on SuperFrost Plus slides (Thermo Fisher) and stored at −20°C until they were processed for immunofluorescent staining.

For immunofluorescent staining, cryosection slides were allowed to reach room temperature and then rehydrated in 1x PBS for 10 min. Samples were surrounded with a ReadyProbes™ Hydrophobic Barrier Pap Pen (Thermo Fisher) and blocked for 1 h at room temperature with PBS + 0.2% Triton-X100 (PBST) + 2% bovine serum albumin (BSA). The samples were washed three times with PBST, each wash lasting 3 min. They were then stained with the following primary antibodies diluted in PBST + 2% BSA overnight at 4°C: 1:500 rabbit anti-MBP (Thermo Fisher, Cat# PA5-78397, Polyclonal) and 1 μg/mL of Rat anti-mouse PD-1 (BioXcell, Clone: RMP1-14, Cat# BE0146). Furthermore, the samples were washed three times with PBST for 3 min and then stained for 1 h at 4°C with 1:400 donkey anti-rabbit AlexaFluor647 (Jackson ImmunoResearch, Cat# 711-605-152) and 1:400 goat anti-rat AlexaFluor488 (Jackson ImmunoResearch, Cat# 112-545-003). The samples were then washed three times with PBST, mounted in fluoroshield mounting medium using DAPI (Abcam, Cat# ab104139), and sealed with nail polish. The samples were analyzed using ZEN software on a Leica LSM 700 confocal microscope at 10× and 20× magnification. Background immunofluorescence and non-specific signal was attributed to the secondary antibody alone during imaging and controls before fluorescent settings.

## Results

### Fingolimod enhances RIT therapy in the MOG^35-55^ EAE model

**Figure 2:**
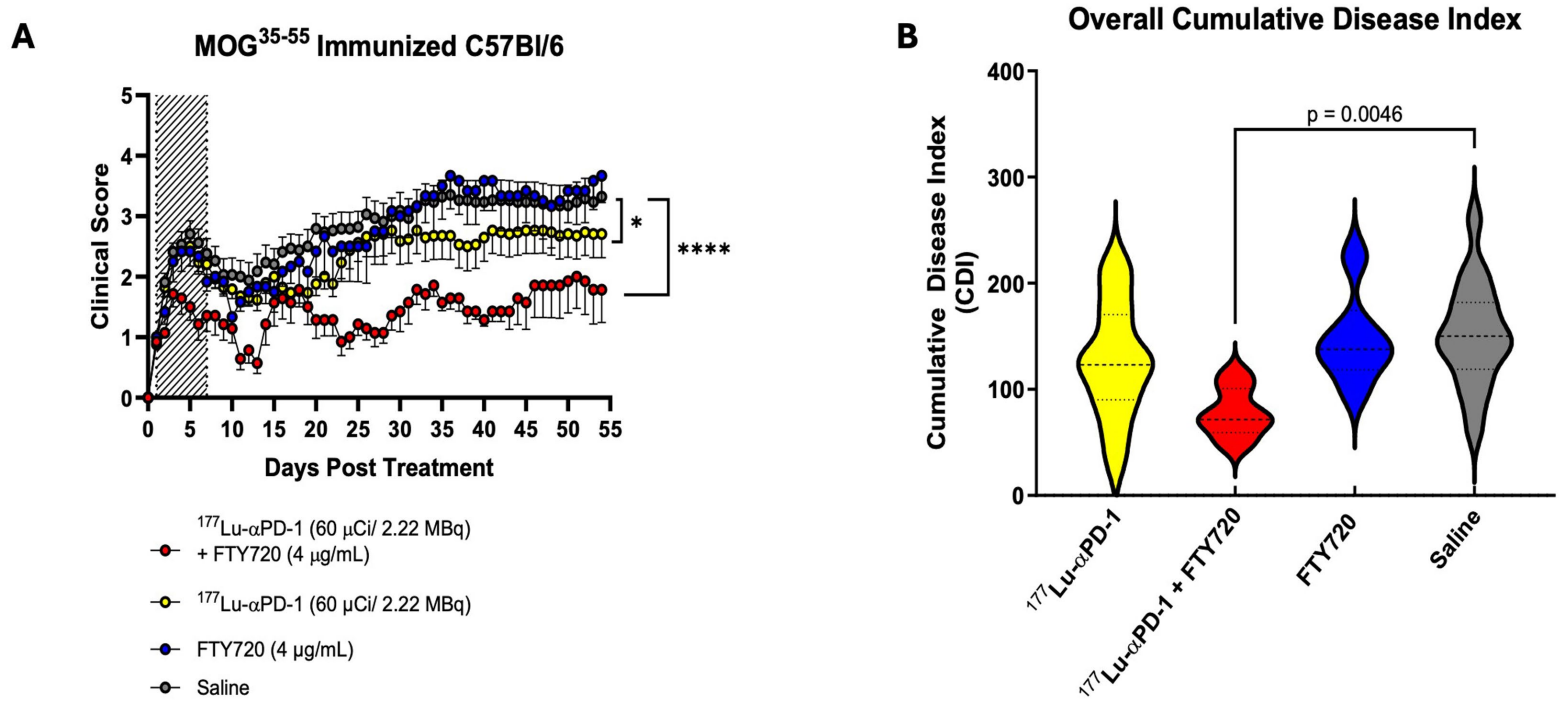
**(A)** Clinical score data of MOG^35-55^ EAE mice treated with combination of short term FTY720 + αPD-1 RIT [n = 7/group (FTY720 alone + FTY720 + αPD-1), n = 17/group (αPD-1 alone + saline)]. Hashed area represents dates of FTY720 administration (Day 0 to 7). Data presented as clinical score ± SEM. Data analyzed via non-parametric Kruskal Wallis Test, *p* < 0.0332 (*), *p* < 0.0021 (**), *p* < 0.0002 (***) and *p* < 0.0001 (****). **(B)** Cumulative disease index (CDI), total clinical score over study period of each group. Data presented as CDI ± SD and analyzed via one-way ANOVA with multiple comparisons.

Animals (n = 17, ^177^Lu-αPD-1 and saline n = 7, ^177^Lu-αPD-1 + FTY720 and FTY720 alone) were immunized, and at symptom onset, they were randomized to receive RIT, RIT + FTY720, FTY720 alone, or saline vehicle. Radiochemical characterization was performed on 177Lu-αPD-1, reflecting >95% radiochemical purity via radio-HPLC and radio-iTLC analysis (Supplementary Figure 3). Animals receiving RIT + FTY720 demonstrated significantly suppressed EAE activity over the study duration (Figure 2A). RIT monotherapy demonstrated moderate suppression of EAE activity. Animals receiving RIT + FTY720 demonstrated a significant reduction in acute paralysis during the first 7 days compared to RIT, FTY720, and saline groups. Short-term FTY720 had no significant effect alone on overall EAE disease progression. A significant reduction in disease was observed in animals treated with ^177^Lu-αPD-1 alone, which was further enhanced by the addition of FTY720. Analysis of the cumulative disease index (CDI), a cumulative measure of daily disease of the entire treatment group over the study period, demonstrated a significant suppression of the severity of disease in animals treated with RIT + FTY720 (Figure 2B).

### RIT and FTY720 combination reduce infiltrating lymphocytes in the spinal cord and minimizes demyelination in chronic MOG^35-55^ EAE

**Figure 3:**
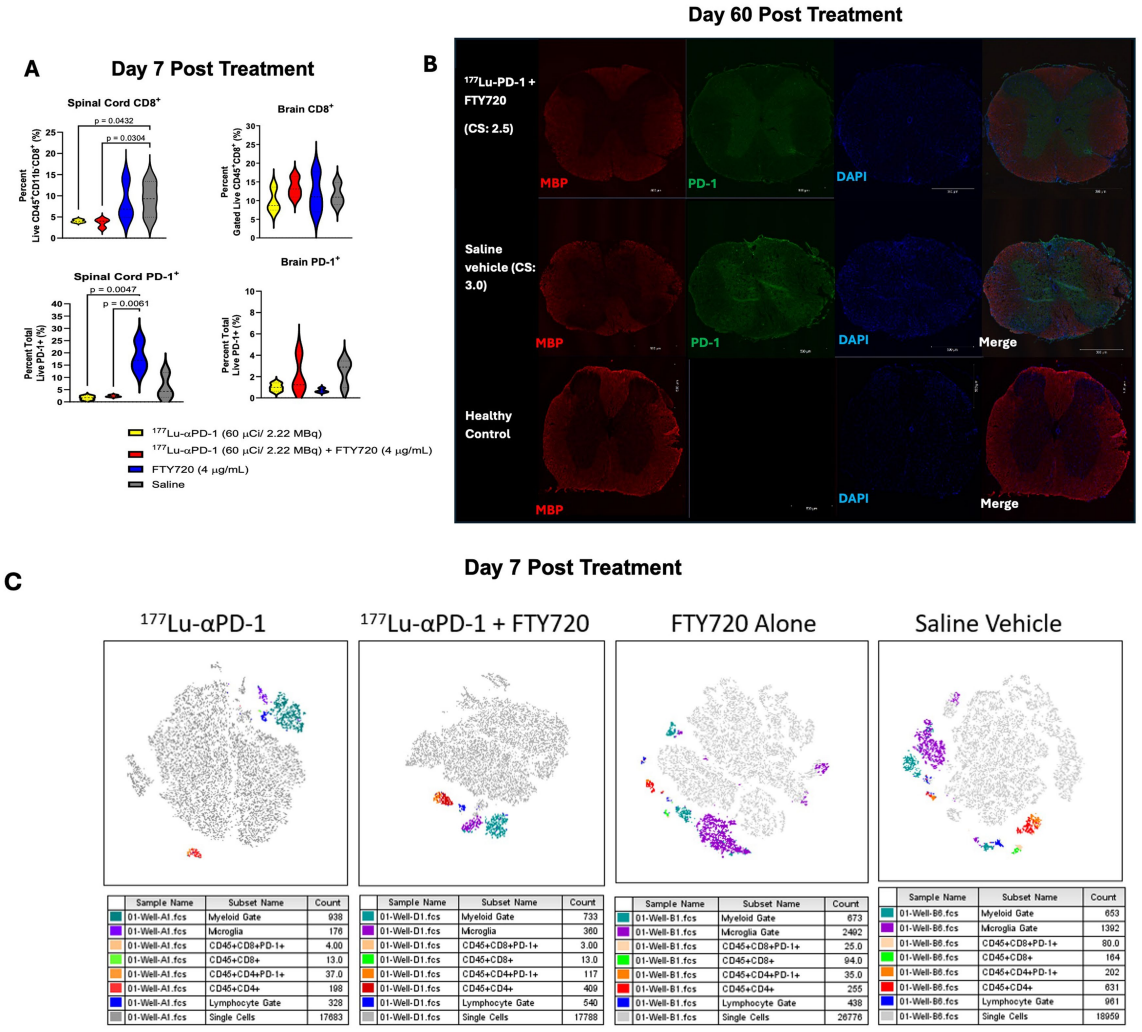
**(A)** Total spinal cord and brain infiltrating CD8^+^ T cells and PD-1+ cells in MOG^35-55^ EAE mice 7 days post treatment (n = 3/group). Data presented as percentage ± SD and was analyzed via two-way ANOVA with *post-hoc* Tukey's multiple comparisons. Significant *p* values reported, where *p* < 0.0332 (*), *p* < 0.0021 (**), *p* < 0.0002 (***) and *p* < 0.0001 (****). **(B)** Immunofluorescent microscopy of spinal cords of mice treated with αPD-1 RIT + FTY720 or saline for MBP, PD-1 and DAPI. C.S., clinical score at time of euthanasia. Healthy control comparison depicted for reference. **(C)** Representative t-SNE (t-distributed stochastic neighbor embedding) maps of spinal cord infiltrating mononuclear cells of ^177^Lu-αPD-1, ^177^Lu-αPD-1 + FTY720, FTY720 alone or saline vehicle treated MOG^35-55^ EAE induced C57B1/6 mice. Gates defined as myeloid (CD11b^+^, CD45^+^), microglia (CD11b^+^, CD45^dim^) and lymphocyte (CD11b^−^, CD45^+^).

To better understand the acute effects of RIT + FTY720, flow cytometry was performed on isolated mononuclear spinal cord cells, which was obtained from animals 7 days post-administration of RIT ± FTY720 (n = 3/group). We observed some deviations in major immune cell subsets in the spleen and the brain at 7 days post-treatment (Supplementary Figure 1). Reduced numbers of CD3^+^ T cells and CD8^+^ T cells were found in the spleens of mice treated with FTY720. No significant differences in the CD4/CD8 T cell ratio were detected in the spinal cords of treated animals. Significant reduction in infiltrating CD8^+^ T cells and total PD-1^+^ cells infiltrating the spinal cord were seen in animals treated with RIT and RIT + FTY720 compared to FTY720 alone or saline (Figure 3A). Surprisingly, PD-1^+^ cells were increased in short-term FTY720 therapy. We aimed to observe whether there were reductions in CD8^+^ T cells and PD-1^+^ cells infiltrating the brain tissue during acute EAE. However, we detected no difference between any of the groups and noted an overall low abundance of PD-1^+^ cells in the brain (<4% of total cells). At study termination, we assessed the long-term effects of RIT + FTY720 therapy on myelin and activated T cells. We performed immunohistochemistry analysis for myelin basic protein (MBP) and PD-1 as a marker for activated T cells (Figure 3B). Representative spinal cord sections of the treated animals demonstrated fewer PD-1^+^ infiltrates than RIT + immunomodulation therapy mice. Single-cell tSNE analysis of flow cytometry data demonstrated increased myeloid and microglial clustering in FTY720 and control-treated animals. RIT and RIT + FTY720 showed changes in lymphocyte clustering and reductions in total CD8^+^ T cells (Figure 3C).

### Fingolimod enhances lymphatic uptake of PD-1-targeted radioimmunoconjugate in MOG^35-55^ EAE mice

**Figure 4:**
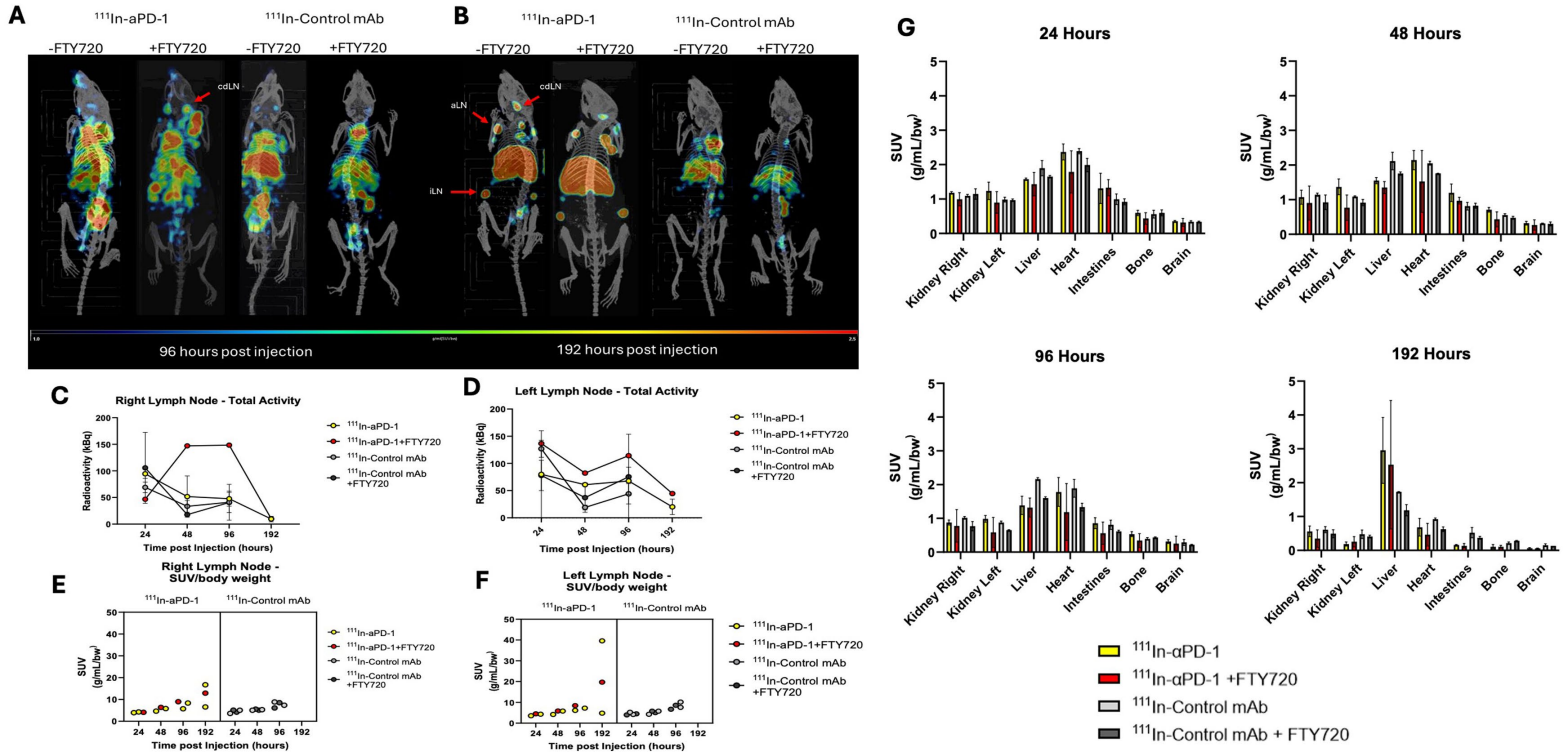
ImmunoSPECT/CT imaging of MOG^35-55^ EAE immunized mice with ^111^In-αPD-1 radioimmunoconjugate ± FTY720 or irrelevant control mAb. **(A)** ImmunoSPECT/CT images at 96 h post injection and **(B)** 192 h post injection. Arrows highlight lymphatic tissue where cdLN, cervical draining lymph node; aLN, axial lymph node; and iLN, inguinal lymph node. Decay correct radioactivity quantification of images for **(C)** right cervical lymph node and **(D)** left cervical draining lymph node. Standardized uptake value/body weight (SUV/bw) corrected values for **(E)** right cervical draining lymph node and **(F)** left cervical draining lymph node. **(G)** Biodistribution data of radiolabeled ^111^In-αPD-1 and ^111^In-Control mAb at 24, 48, 96 and 192 h post injection.

Finally, to confirm targeting and uptake in lymphatic tissue as a synergistic mechanism with FTY720, we performed immunoSPECT/CT imaging of MOG^35-55^ immunized mice administered ^111^In-αPD-1 ± FTY720. Animals (n = 2/group, n = 1 PD-1 + FTY720) were injected at symptom onset and serially imaged at 24, 48, 96, and 192 h post-injection of radiotracer. A second irrelevant control antibody was given separately to confirm specific uptake of the αPD-1 antibody compared to a non-specific antibody. Early timepoints (24 and 48 h) demonstrated systemic circulation of the antibody (Supplementary Figure 2), while specific accumulation in lymphatic tissue began to materialize by 96 h and was observed at 192 h (Figure 4A). FTY720 enhanced the uptake of αPD-1 antibody at 96 h post-injection, with 192 h showing similar uptake in cervical and inguinal lymph nodes with or without FTY720. Standardized uptake value (SUV/bw) and analysis of left and right cervical draining lymph nodes revealed similar increasing retention over time, but an increased amount of calculated radioactivity (kBq) in the presence of FTY720 in the right lymph node (Figures 4C–F). Low accumulation of the irrelevant control IgG in lymphatic tissue with or without FTY720 was seen (Figure 4B). Biodistribution of radioimmunoconjugates demonstrates the distribution of IgGs in the kidneys, liver, and heart. The clearance of radiolabeled IgGs by 192 h is visible through the accumulation of radioactivity in the liver (Figure 4G).

## Discussion

Long-term treatment of MS is currently managed by various immunomodulatory and immunosuppressive DMTs that target immune cells such as B cells and T cells, which are associated with disease progression. FTY720 is a water-soluble immunomodulatory agent that has demonstrated efficacy in EAE through oral administration and injection. FTY720 has strong oral bioavailability in animals and is readily absorbed into the lymphatic tissue (23). Groups have demonstrated therapeutic benefits in the MOG^35-55^ EAE model through oral and intraperitoneal administration of FTY720 (24, 25). Exposure of FTY720 to animals through drinking water (0.5 mg/kg/day) increased survival in heart allograft models and restricted lymphocyte trafficking in viral infection models (26, 27). We decided to administer FTY720 through drinking water to reduce restraint stress, a factor known to suppress EAE development in rats and mice (28).

We sought to establish a T cell-targeted therapy that specifically targets activated cells through the PD-1 receptor, compared to a pan-T cell ablation strategy that may suppress disease but lead to increased immunodeficiency. PD-1 has been demonstrated to have a key role in EAE pathogenesis, and blockade of PD-1 signaling induces severe disease and death in MOG^35-55^ EAE mice (12). Our approach utilizes PD-1 as a targeting moiety in contrast to inhibiting its signaling. Selective ablation of autoreactive cells is possible by utilizing PD-1 as a marker for activated and chronically TCR-stimulated lymphocytes, selective ablation of autoreactive cells is possible. We did not observe severe paralysis and death in our models, owing to the low amounts of compound injected (6 μg) in contrast to previous PD-1 blocking studies utilizing 500 μg of blocking (J43 clone) PD-1 antibody injected intraperitoneally. This highlights the advantage of utilizing high potency and low mass quantities of radioimmunoconjugates as therapeutic tools.

We previously established and optimized the radiochemical synthesis and therapeutic performance of this ^177^Lu radioimmunoconjugate targeted to PD-1 in the EAE model. We previously observed retention of the radioimmunoconjugate in the lymph nodes of EAE mice during nuclear imaging. We hypothesized that FTY720 may further enhance the therapeutic performance of PD-1-targeted radioimmunotherapy through the entrapment of pathogenic T cells within the lymph nodes, allowing for concentrated irradiation. RIT monotherapy demonstrated moderate suppression of EAE activity. Animals receiving RIT + FTY720 significantly reduced acute paralysis for the first 7 days compared to RIT, FTY720, and saline treatments. Surprisingly, PD-1^+^ cells were increased in short-term FTY720 therapy.

This result may align with FTY720 withdrawal syndrome and an increased release of activated PD-1^+^ cells from the lymph node after FTY720 discontinuation (29). The retention of the control antibody at the emulsion site could be partly explained by enhanced blood flow and/or non-specific Fc binding of dendritic cells and macrophages to the control IgG (30). The outcomes of this study support the hypothesis that FTY720 significantly enhanced the performance of a T cell-targeted radioimmunotherapy in EAE. We observed no effect of non-radiolabeled anti-PD-1 control in the EAE model as previously described (21).

Radiopharmaceutical agents are increasingly becoming an attractive option as dual therapeutic and diagnostic agents. Theranostic radiopharmaceuticals can allow visualization of disease activity during treatment. Clinically relevant imaging modalities of neuroinflammation and immune cell activity could aid in guiding therapy and monitoring responses in MS patients. Here, we demonstrated successful visualization of disease activity in MOG^35-55^ - mediated inflammation via PD-1 antibody binding, presumably corresponding to T cell activation in the lymph nodes of mice. The performance of a full-length IgG vector, such as the one utilized in this study, could be optimized further to enhance CNS tissue penetration and permeability through the blood–brain barrier. Obstacles exist wherein full-length IgG is limited in its accessibility to CNS tissue (31). In the case of EAE, after successfully infiltrating immune cells into the CNS during the early stages, targeting therapy of pathogenic cells using IgG may become difficult. In these studies, animals were treated at symptom onset, which we have previously demonstrated correlates with a vast infiltration of immune cells into the spinal cord. Therefore, modification of targeting vectors to PD-1, such as nanobodies (VHH), fragment antibodies (Fab’), single chain variable fragments (scFv), or modification with BBB permeable peptides may allow for increased therapeutic potential of PD-1 radiopharmaceutical therapy (32–35). This would be partly due to increased permeability to CNS tissue and binding to PD-1^+^ pathogenic T cells. Improved clearance and lower retention in the liver, as observed with full-length IgGs, may also optimize the safety profile of T cell-targeted RIT through reduced total body absorbed dose of radiation. Further evaluation of optimized vectors for the delivery of RIT to activated T cells in autoimmune disease could be a promising avenue for translatable therapy.

RIT offers many clinical advantages, including minimal risk of immunoreactivity due to reduced concentrations of drug and highly potent and specific cytocidal delivery to target cells. Concerns about using radioisotopes therapeutically are possible through the generation of DNA strand breaks and off-target killing of non-targeted cells via bystander effect or absorption of radiation near targeted cells (36). In our case, care should be taken to evaluate the hematological and immunological effects of RIT due to the observation of enhanced uptake in lymphatic tissue. White blood cells and T lymphocytes are typically prone to radiation-induced cell death (37). This is an advantage as the driving factor of disease in EAE and MS is immune-mediated, but the risk of immunosuppression via irradiation is higher. Increased irradiation of lymphatic tissue via uptake of PD-1 RIT is likely to reduce T cell and potentially B cell function, which has yet to be fully elucidated. Macrophage and dendritic cells (DCs) are known to be remarkably resistant to radiation due to the presence of internal machinery necessary to degrade proteins (38, 39). Both cell types possess phagolysosomes to promote the generation of reactive oxygen species (ROS) and proteolytic enzymes to process exogenous proteins into peptides before presentation through MHC complexes. These functions have evolved to allow macrophages and DCs to generate defense mechanisms to protect against ROS, likely resulting in increased resistance to irradiation (40, 41). Further complications in predicting the outcome of RIT on lymph node function are the observations that radiation possesses immunomodulatory effects on both macrophages and DCs (38, 42, 43). RIT targeting immune cells such as T cells may induce immunomodulatory effects via non-specific irradiation of DC and macrophages in proximity in lymphatic tissue. However, these studies are often undertaken from gamma, proton, or x-ray irradiation of cancer and analysis of macrophage and DC function thereafter, which may not reflect the response of these cells in the context of targeted beta particle irradiation as described in this study. Further evaluation of immune function post-RIT is an important and necessary step to promote clinical translatability of RIT for autoimmune disease. RIT may be a potential avenue to ablate pathogenic T cells in combination with FTY720, and allow patients to potentially remove the need for indefinite FTY720 treatment with long-term stabilization of T cell autoreactivity.

In conclusion, we present validation of a PD-1 targeting radiolabeled antibody as a therapeutic and imaging agent in MOG^35-55^-induced EAE. We demonstrated that enhanced disease suppression was achieved with short-term FTY720 therapy, which increased lymph node retention of the radiopharmaceutical agent and reduced infiltrating CD8^+^ T cells during the early stages of disease. These findings suggest that targeted irradiation of T cells using a PD-1-targeted radioimmunoconjugate can be applied in combination with FTY720, an already approved immunomodulatory agent for MS. Theranostic nuclear medicine approaches such as the one described in this study could be a useful tool clinically for the treatment and monitoring of T cell activity in patients suffering from T cell-mediated autoimmune diseases, such as MS.

## Data Availability

The raw data supporting the conclusions of this article will be made available by the authors without undue reservation.
