# Supplementary material for: Fingolimod synergizes with anti-PD-1 radioimmunotherapy in an experimental multiple sclerosis model through enhanced lymph node retention and CD8+ T cell depletion
Source: Front Med (Lausanne). 2025 Jul 3;12:1593933. doi: 10.3389/fmed.2025.1593933 (PMC12267204; doi:10.3389/fmed.2025.1593933)
Supplement: Supplementary file 1 [file Data_Sheet_1.pdf]

Supplemental Data: Synergistic Effects of Fingolimod and anti-PD-1 radioimmunotherapy in an experimental multiple sclerosis model through enhanced lymph node retention and CD8+ T cell depletion

Frank, C.[1], Allen, K.J.H.[1], Dawicki, W.[2], Levin, M.C.[3,4,5,6], Dadachova, E[1]*.

[1]College of Pharmacy and Nutrition, University of Saskatchewan, Saskatoon, SK, S7N 5E5, Canada.

[2]Department of Biochemistry, Microbiology and Immunology, College of Medicine, University of Saskatchewan, Saskatoon, SK, S7N 5E5, Canada

[3]Office of the Saskatchewan Multiple Sclerosis Clinical Research Chair, University of Saskatchewan, Saskatoon, SK, S7K 0M7, Canada

[4]Cameco MS Neuroscience Research Centre, College of Medicine, University of Saskatchewan, Saskatoon, SK, S7K 0M7, Canada

[5]Neurology Division, College of Medicine, University of Saskatchewan, Saskatoon, SK, S7N 0X8, Canada

[6]Department of Anatomy, Physiology and Pharmacology, College of Medicine, University of Saskatchewan, Saskatoon, SK, S7N 5E5, Canada

*Corresponding Author

ekaterina.dadachova@usask.ca

+13069665163

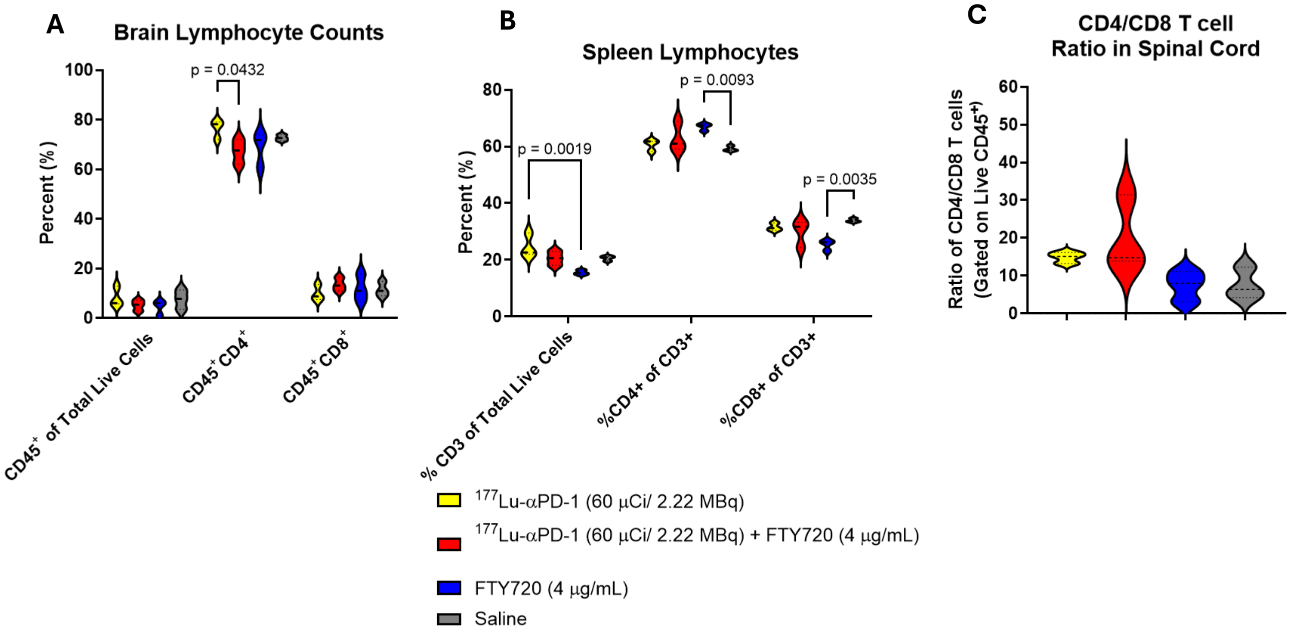

Supplemental Fig 1. Total A) brain and B) spleen counts of $CD3^+$, $CD4^+$ T cells and $CD8^+$ T cells in MOG^{35-55} EAE 7 days post treatment with aPD-1 RIT (n=3/group). Data analyzed via Two-way ANOVA with Tukey's multiple comparisons. C) Ratio of $CD4^+/CD8^+$ T cells within the spinal cord of MOG^{35-55} EAE treated mice.

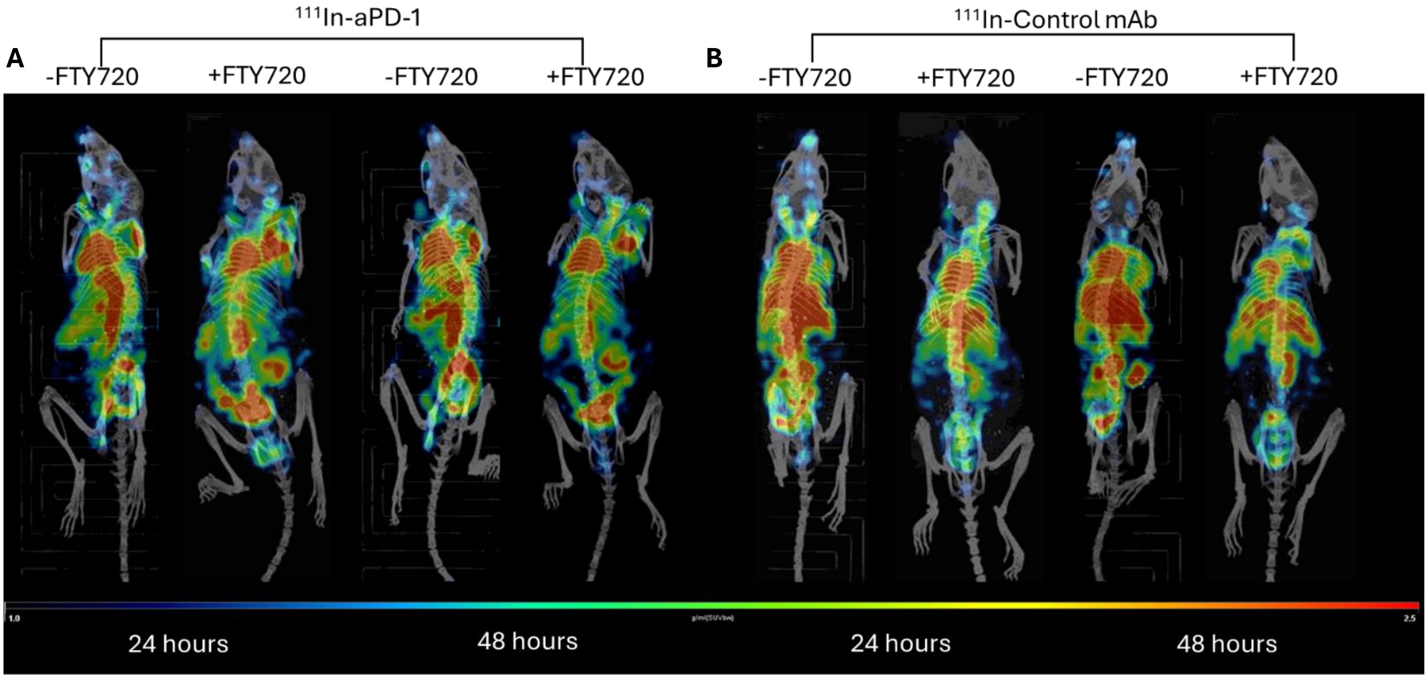

Supplemental Fig 2. Systemic circulation of radiolabeled antibodies in MOG^{35-55} EAE mice at 24 and 48 hours post injection. A) ^{111}In-αPD-1 circulation ± FTY720. B) ^{111}In-control mAb ± FTY720. Data presented on a scaled range of 1.0 to 2.5 SUV/bw.

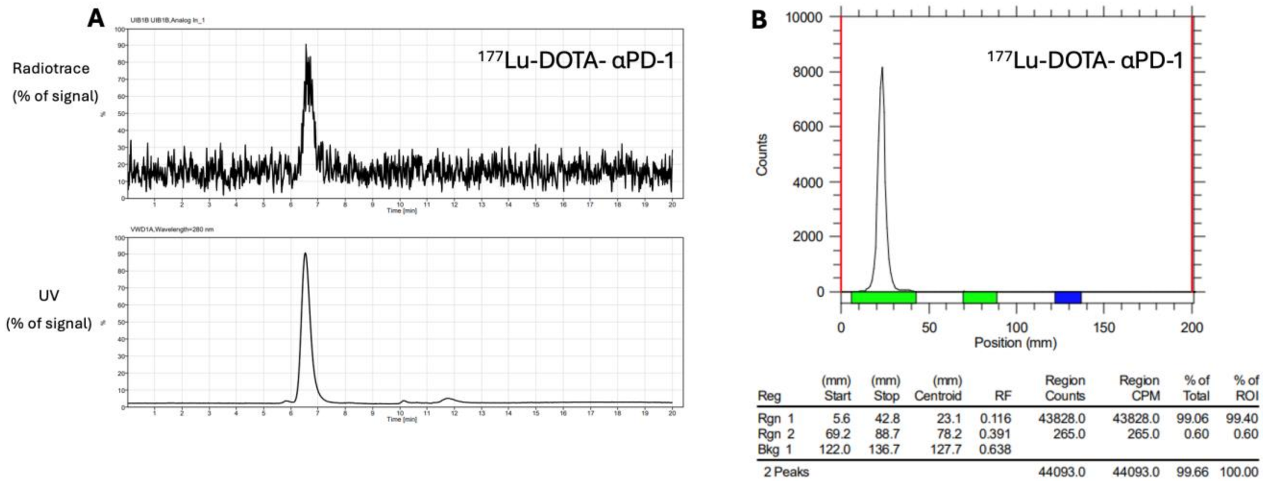

Reg	(mm) Start	(mm) Stop	(mm) Centroid	RF	Region Counts	Region CPM	% of Total	% of ROI
Rgn 1	5.6	42.8	23.1	0.116	43828.0	43828.0	99.06	99.40
Rgn 2	69.2	88.7	78.2	0.391	265.0	265.0	0.60	0.60
Bkg 1	122.0	136.7	127.7	0.638				
2 Peaks					44093.0	44093.0	99.66	100.00

Supplemental Fig 3. Radiochemical characterization of ^{177}Lu-DOTA- αPD-1 (^{177}Lu-αPD-1) via A) Radio-HPLC and B) Radio-iTLC. All ^{177}Lu and ^{111}In radiolabeling yields of DOTA-aPD-1 exceeded 95% radiochemical purity.
